# Comparing the Association Between Dementia Risk Scores and Cognitive Function Among Members of a Research‐based Community Centre for Dementia Risk Reduction

**DOI:** 10.1002/alz70860_098233

**Published:** 2025-12-23

**Authors:** Danielle D'Amico, Brian Tan, Deanise Berba, Howard Chertkow, Nicole D. Anderson

**Affiliations:** ^1^ Baycrest Academy for Research and Education, Toronto, ON, Canada; ^2^ University of Toronto, Toronto, ON, Canada

## Abstract

**Background:**

Dementia risk scores may be useful in facilitating communication of risk to adults seeking to support their health and wellness, maintain their cognitive function, and reduce their risk of developing dementia. While various scoring algorithms exist, limited research has compared how these scores are differentially associated with cognition among community‐dwelling adults.

**Method:**

The objective of this study was to examine the association between five dementia risk scores (CogDrisk, LIBRA, modified LIBRA [mLIBRA], CAIDE, and Brain Care Score [BCS]) and cognitive function among 253 adults without dementia who are members of a research‐based community centre for dementia risk reduction (mean age = 69.6±8.9 [range = 50 ‐ 95], 77% female). Cognitive function was assessed using Cogniciti's Brain Health Assessment (BHA) total score, with higher scores representing better cognition. Higher dementia risk scores indicate greater dementia risk, with the exception of the BCS. Generalized linear models were used to determine the associations between dementia risk scores and cognitive function.

**Result:**

Lower CogDrisk scores (*r* = ‐0.36, *p* < 0.001), mLIBRA scores (*r* = ‐0.37, *p* < 0.001), and CAIDE scores (*r* = ‐0.15, *p* = 0.02) were associated with higher BHA scores. LIBRA scores (*r* = ‐0.08, *p* = 0.21) and BCS scores (*r* = 0.07, *p* = 0.28) were not significantly associated with BHA scores.

**Conclusion:**

Findings suggest scoring algorithms that include non‐modifiable sociodemographic information (age and sex) have a stronger association with cognitive function compared to those that only include modifiable factors (e.g., physical activity, hypertension, diabetes). As this study was cross‐sectional in nature, future research should examine how baseline and change in dementia risk scores predict change in cognitive function over time in order to better guide implementation.

